# Editorial: A New Era in Dentistry: Stem Cell-Based Approaches for Tooth and Periodontal Tissue Regeneration

**DOI:** 10.3389/fphys.2016.00357

**Published:** 2016-08-19

**Authors:** Thimios A. Mitsiadis, Giovanna Orsini

**Affiliations:** ^1^Orofacial Development and Regeneration, Centre for Dental Medicine, Institute of Oral Biology, University of ZurichZurich, Switzerland; ^2^Department of Clinical Sciences and Stomatology, Polytechnic University of MarcheAncona, Italy

**Keywords:** tooth, dentin, enamel, stem cells, scaffolds, vasculature, craniofacial, regenerative dentistry

The continuously increasing progress in stem cell biology has expanded our horizons into the principles of health and disease. This new acquired knowledge and the recent advances in the field of biotechnology have formed the substrate for translation into novel therapeutic solutions (Mitsiadis et al., 2015). Indeed, traditional dental therapies have successfully incorporated a good number of new materials that are more compatible and friendly to dental and periodontal tissues. Although these treatments are suitable and satisfactory for long-lasting dental restorations, the idea of stem cell-based tissue regeneration is tempting and represents the decisive objective of introducing new therapeutic approaches in the clinics. Teeth develop through continuous and reciprocal interactions between cranial neural crest-derived mesenchymal stem cells and oral-derived epithelial stem cells during early embryogenesis (Mitsiadis and Graf, 2009; Smith et al., 2009; La Noce et al., 2014). These highly mineralized organs are composed by collagenous (such as the mesenchyme-derived dentin and cementum structures) and non-collagenous (such as the epithelium-derived enamel structure) extracellular matrices. The alveolar bone is another collagenous hard tissue, which through its close interaction with the periodontal ligament supports tooth stability and function. Tooth vitality is ensured by blood vessels and nerve fibers within the dental pulp and periodontium (Carmeliet and Jain, 2011; Pagella et al., 2015). Regenerative approaches include the formation of new enamel, dentin, pulp, periodontal ligament, and alveolar bone after tooth damage due to genetic pathology, traumatic injuries, caries, and periodontal lesions (Mitsiadis and Harada, 2015). Therefore, the recent progress made in the fields of stem cell biology, tissue engineering, and nanotechnology offers promising opportunities to repair damaged or missing dental tissues (Mitsiadis et al., 2012). Several considerable efforts have been made recently in the dental field and new concepts concerning dental treatment strategies have arisen with the hope to translate basic research findings into the clinics. Repairing teeth by autologous cell grafting is highly desired, but some ethical, economic, and legislative issues should be first resolved.

In this research topic, prominent researchers within the dental field provided important information, gave responses, and generated stimulating hypotheses concerning the development and regeneration of dental and periodontal tissues. A balanced mosaic of original and review articles point out different facets of the interplay between stem cell biology and dental tissue formation, homeostasis, and regeneration.

The different sources of stem cells, their differentiation potential, and the current state of stem cell-based strategies for dental or craniofacial tissue regeneration are discussed in several articles. The cranial neural crest-derived dental stem cells or stem cells from other tissues can be used with success for dental, periodontal, and craniofacial repair purposes (Akita et al.; Aurrekoetxea et al.; Ducret et al.). The appropriate selection of stem cells in conjunction with the creation of an adequate and respective microenvironment is essential for a successful clinical outcome. Therefore, the identification and characterization of stem cell niches within dental tissues, and the analysis of niche-derived signals during tooth homoeostasis and repair is of prime importance (Oh and Nor). The exploitation of various adult stem cell populations localized in the different tooth components is challenging. Furthermore, success depends on well-controlled and coordinated cytodifferentiation and mineralization processes during tissue repair and regeneration (Eapen and George).

Small-scale engineered biomaterials and tailor made empty scaffolds that will attract specific endogenous stem cell populations can be designed for the regeneration the periodontium (Green et al.) as well as of various tissues within the craniofacial complex (Mele et al.). Innervation of craniofacial tissues, including teeth, is essential for ensuring their morphology, physiological function, and regeneration (Fried and Adameyeko). Equally, blood supply of craniofacial structures and teeth are important for their function, vitality, and longevity, and therefore it is important to develop methods for analyzing angiogenesis in regenerating tissues (Woloszyk et al.). One of these methods allowed us to evaluate and compare the ability of human dental pulp stem cells and gingival fibroblasts in attracting vessels into silk fibroin scaffolds (Woloszyk et al.). The tissue-engineering concept for dental pulp regeneration implicates stem cells, scaffolds, and signaling molecules (Passos et al.; Yang et al.). Pulp-derived stem cells have the potential to differentiate into odontoblasts and other cell types and thus could be used for clinical applications. Odontoblasts produce nitric oxide with antibacterial activity, which is an important issue for successful tissue regeneration purposes (Farges et al.). During tooth homeostasis and pathology, communication between odontoblasts is activated via specific channels (Sato et al.). Hydrogels may be used as stem cell-seeded matrices for pulp and periodontal tissue repair purposes in the clinics (Teti et al.). Polymerized resin-based materials could affect cellular physiology and induce cell apoptosis (Teti et al.).

Several pathologies exist that lead to root malformations and consequently affect tooth eruption or/and physiology (Luder). Tooth eruption is a complicated event that can differ from species to species (Liu et al.). Resorption of the alveolar bone that surrounds the tooth root could be an actor of periodontal development and remodeling (Gama et al.). Orthodontic forces affect alveolar bone remodeling and periodontal ligament regeneration through defined molecular mechanisms (Suttorp et al.). The use of biomimetically enhanced demineralized bone matrices could represent an interesting approach for bone regenerative applications (Ravindran et al.). However, the outcome of tissue engineering strategies for bone formation depends on the origin and the culture conditions of the applied stem cells (Mattioli-Belmonte et al.; Spina et al.).

Amelogenin, one of the most important organic elements of enamel, is re-expressed during dental pathology and repair, indicating that it can act as a signaling molecule for dental tissue repair events (Mitsiadis et al.). Furthermore, injection of stem cells at dental pathological sites may enhance the possibility of *de novo* enamel formation. For testing this hypothesis a mouse model was used for the injection of epithelial stem cells derived from the continuously growing incisors (Orsini et al.).

Besides many open scientific questions, strategic concerns also need to be addressed. Controversies in scientific data, opinions, and formulated hypotheses set the starting point for additional research that will benefit to the dental field. Repair and regeneration of dental tissues using stem cell-based approaches combined with new biotechnological tools is an exciting and highly relevant area of research. Further evaluation of existing tools is needed in order to optimize future therapeutic strategies in dentistry. The time for applying novel regenerative therapies in dental clinics has come, thus designing a new era, full of excitement and expectations.

## Author contributions

The authors contributed to the writing, reading, and editing of the present editorial.

### Conflict of interest statement

The authors declare that the research was conducted in the absence of any commercial or financial relationships that could be construed as a potential conflict of interest. The reviewer VD and handling Editor declared their shared affiliation, and the handling Editor states that the process nevertheless met the standards of a fair and objective review.
